# Supplementary material for: Distinct transcriptomic profiles in children prior to the appearance of type 1 diabetes-linked islet autoantibodies and following enterovirus infection
Source: Nat Commun. 2023 Nov 22;14:7630. doi: 10.1038/s41467-023-42763-9 (PMC10665402; doi:10.1038/s41467-023-42763-9)
Supplement: Supplementary file 1 — Supplementary information [file 41467_2023_42763_MOESM1_ESM.pdf]

Distinct transcriptomic profiles in children prior to the appearance of type 1 diabetes-linked islet autoantibodies and following enterovirus infection

Jake Lin[1,2,3,4*], Elaheh Moradi[1,5*], Karoliina Salenius[1*], Suvi Lehtipuro[1], Tomi Häkkinen[1], Jutta E. Laiho[6], Sami Oikarinen[6], Sofia Randelin[1], Hemang M. Parikh[7], Jeffrey P. Krischer[7], Jorma Toppari[8,9], Åke Lernmark[10], Joe Petrosino[11], Nadim J. Ajami[12], Jin-Xiong She[13], William A. Hagopian[14,15], Marian J. Rewers[16], Richard E. Lloyd[11], Kirsi Rautajoki[1+], Heikki Hyöty[6,17+], Matti Nykter[1,18+] on behalf of the TEDDY Study Group

[1]Prostate Cancer Research Center, Faculty of Medicine and Health Technology, Tampere University and Tays Cancer Centre, Tampere, Finland, [2]Biostatistics, Health Sciences, Faculty of Social Sciences, Tampere University, Tampere, Finland, [3]Finnish Institute of Molecular Medicine, FIMM, University of Helsinki, Helsinki, Finland, [4]Department of Medical Epidemiology and Biostatistics, Karolinska Institute, Stockholm, Sweden, [5]A.I. Virtanen Institute for Molecular Sciences, University of Eastern Finland, Kuopio, Finland, [6]Department of Virology, Faculty of Medicine and Health Technology, Tampere University, Tampere, Finland, [7]Health Informatics Institute, Morsani College of Medicine, University of South Florida, Tampa, FL, United States, [8]Research Centre for Integrative Physiology and Pharmacology, Institute of Biomedicine, and Centre for Population Health Research, University of Turku, Turku, Finland, [9]Department of Pediatrics, Turku University Hospital, Turku, Finland, [10]Department of Clinical Sciences, Lund University CRC, Skåne University Hospital, Malmö, Sweden, [11]Alkek Center for Metagenomics and Microbiome Research, Department of Molecular Virology and Microbiology, Baylor College of Medicine, Houston, TX, United States, [12]Program for Innovative Microbiome and Translational Research, Department of Genomic Medicine, University of Texas MD Anderson Cancer Center, Houston, Texas, United States, [13]Jinfiniti Precision Medicine, Inc., Augusta, GA, United States, [14]Pacific Northwest Research Institute, Seattle, WA, United States, [15]Department of Medicine, University of Washington, Seattle, WA, United States, [16]Barbara Davis Center for Childhood Diabetes, University of Colorado, Aurora, CO, United States, [17]Fimlab Laboratories, Pirkanmaa Hospital District, Tampere, Finland, [18]Foundation for the Finnish Cancer Institute, Helsinki, Finland

*these authors contributed equally [+]Co-corresponding authors

Supplementary Information

This PDF contains:

Supplementary Figures 1–17
Supplementary Note 1: Members of the TEDDY Study Group

Other Supplementary Material Includes:

Supplementary Data 1–15

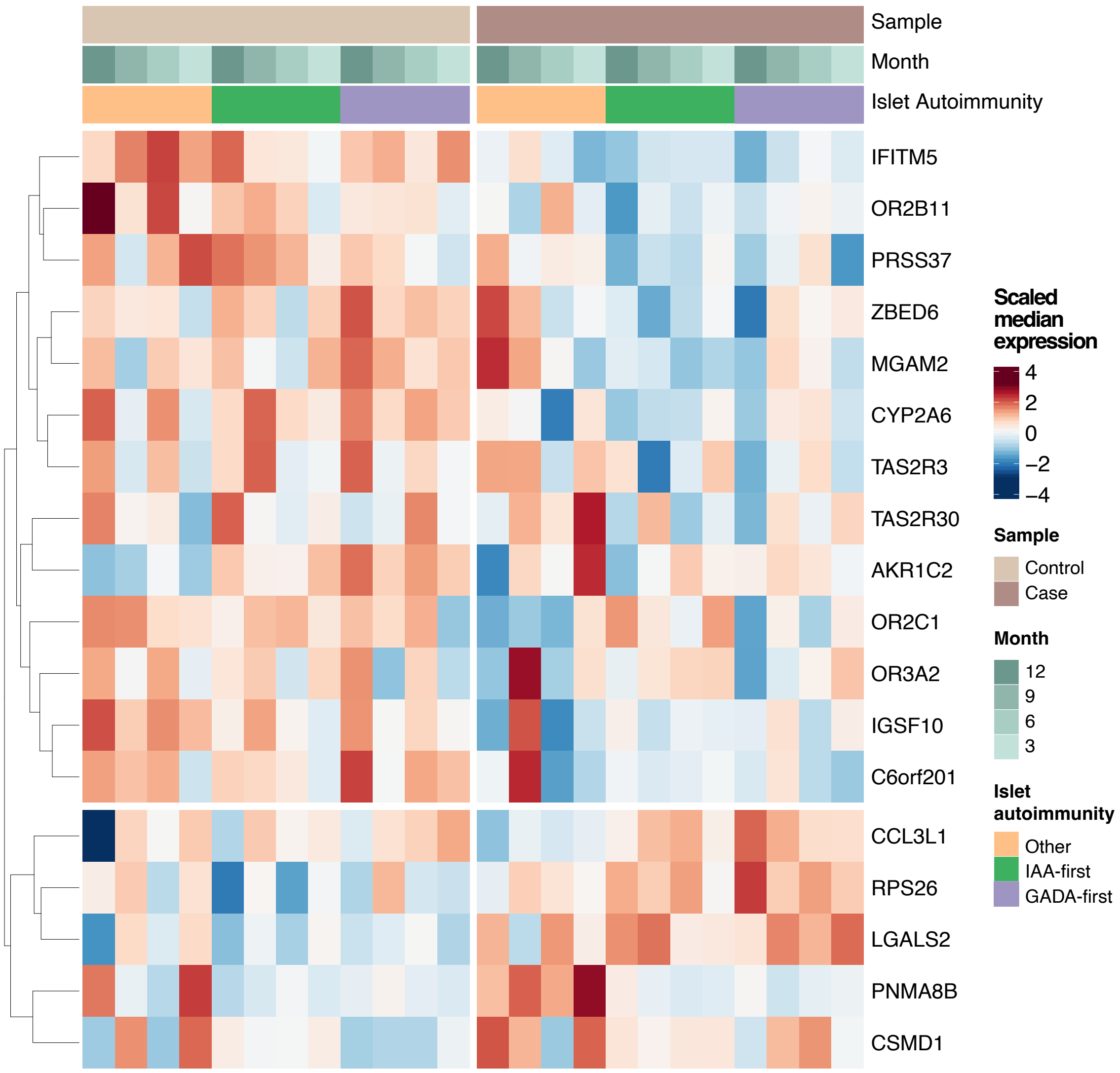

Supplementary Fig. 1: Differential gene expression in Islet Autoimmunity
Heatmap of the differentially expressed genes selected in the islet autoimmunity NCC1 cohort shows their expression across GADA-first, IAA-first and other T1D islet autoimmunity patterns (Supplementary Data 2). Heatmap value is the scaled median expression level at each timepoint.

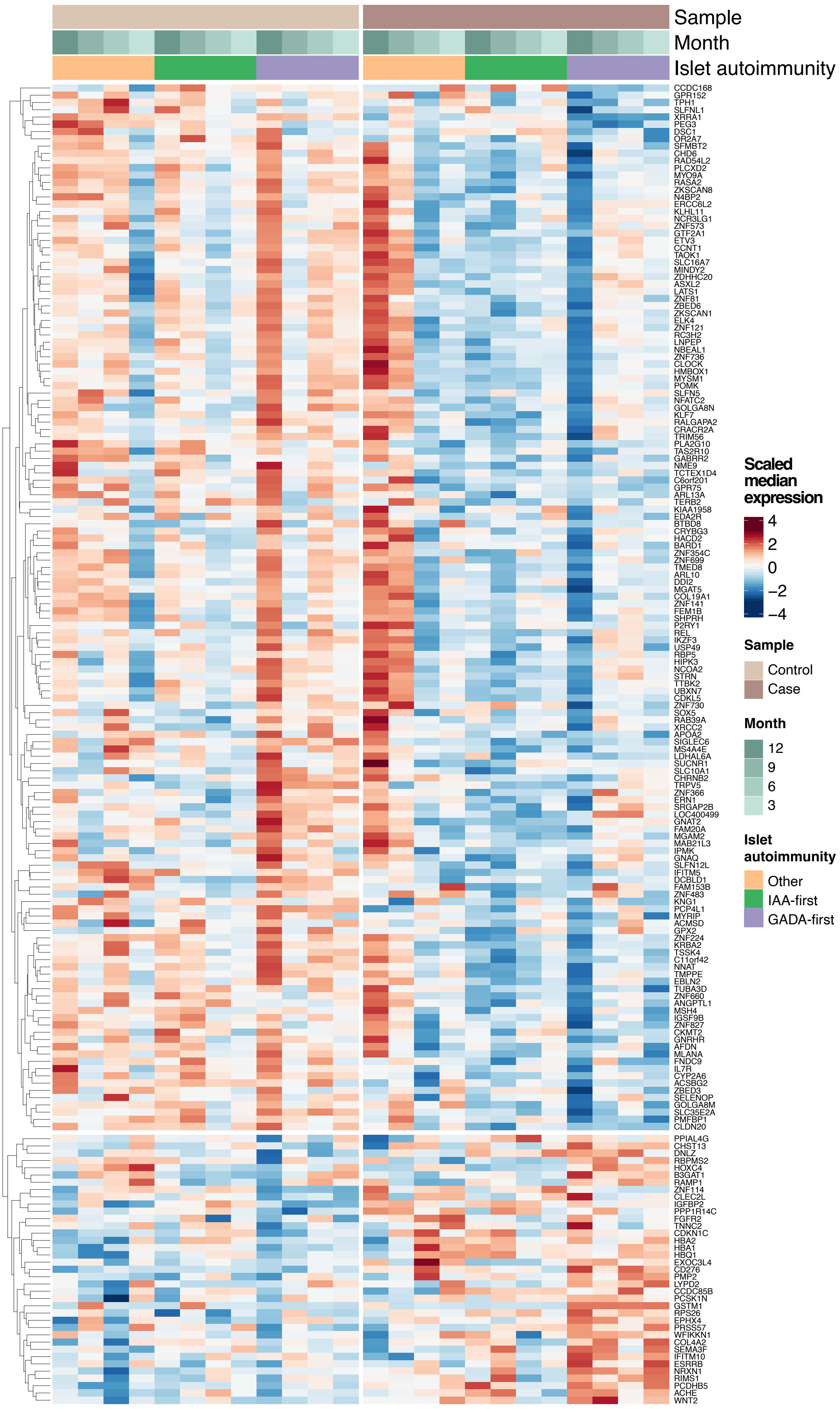

Supplementary Fig. 2: Differential gene expression in GADA-first

Heatmap of the differentially expressed genes selected in GADA-first T1D islet autoimmunity NCC1 cohort shows their expression across GADA-first, IAA-first and other T1D islet autoimmunity patterns (Supplementary Data 2). Heatmap value is the scaled median expression level at each timepoint.

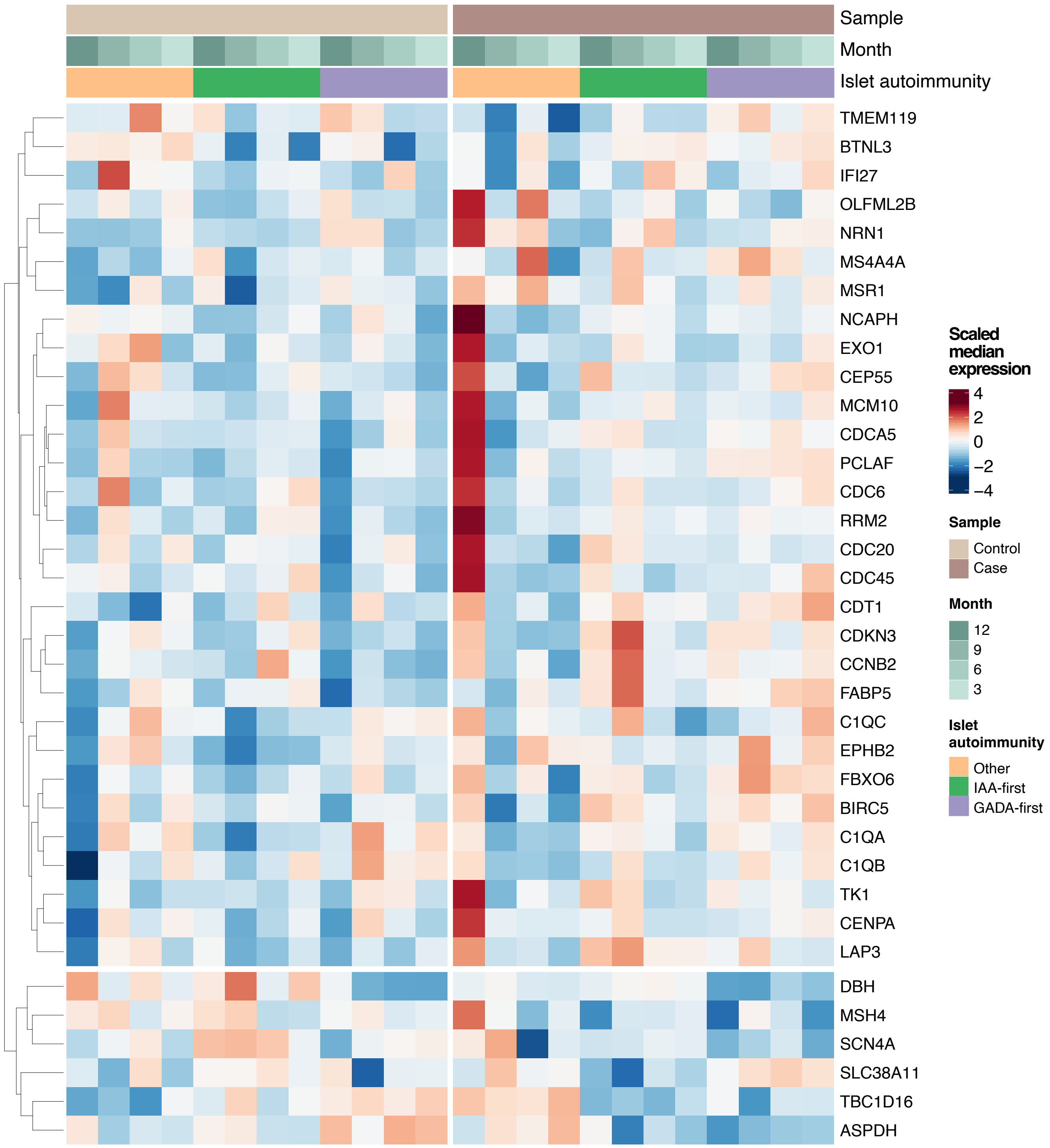

Supplementary Fig. 3: Differential gene expression in IAA-first
Heatmap of the differentially expressed genes selected in IAA-first T1D islet autoimmunity pattern shows their expression across GADA-first, IAA-first and other T1D islet autoimmunity patterns (Supplementary Data 2). Heatmap value is the scaled median expression level at each timepoint.

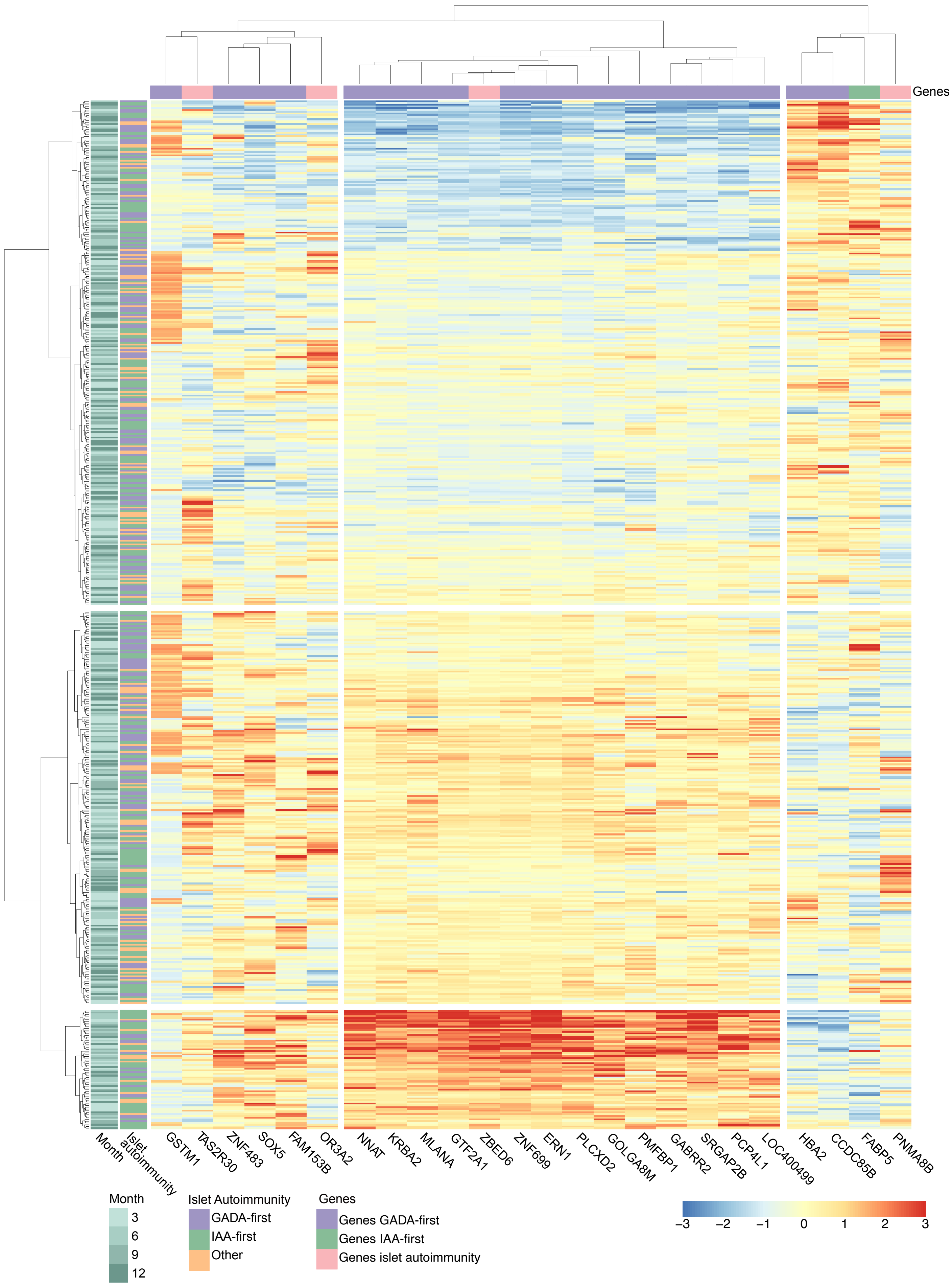

Supplementary Fig. 4: Differential gene expression in genes selected with additional criteria of temporal changes

Distinct endotype GADA-first and IAA-first clusters are formed using the genes selected from the temporal analysis with inclusion of slope changes between samples prior to seroconversion. These genes and their statistical details are shown in Supplementary Data 4. Heatmap value is the scaled ratio between matched case and control.

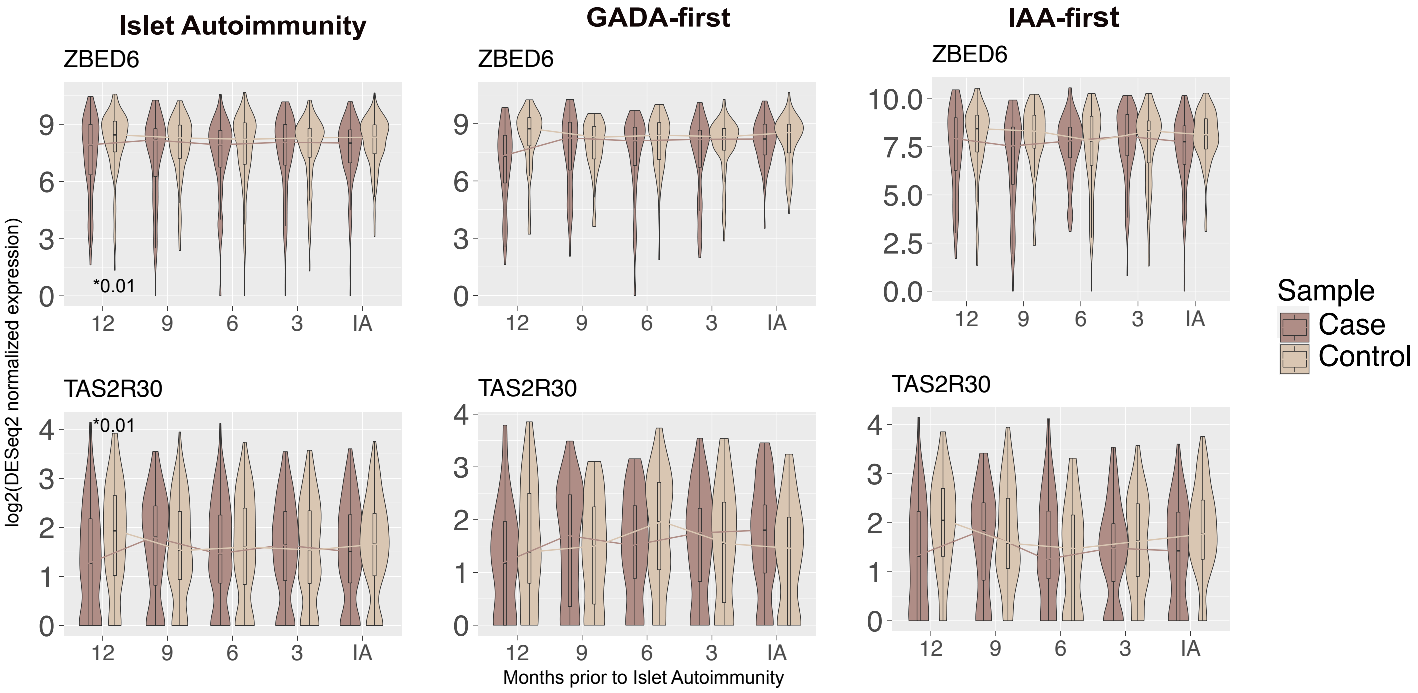

Supplementary Fig. 5: Temporal expression of the genes selected with additional criteria in Islet Autoimmunity

Violin plots of the selected differentially expressed genes with inclusion of temporal slope changes and adjustment for multiple timepoints from IAb conversion to 12 months prior relative to all 3 islet autoimmunity patterns. Figure shows two genes selected with the full islet autoimmunity in all three cohorts (full islet autoimmunity (n=168), GADA 1st (n=55) and IAA first (n=85)) and the corresponding significant p-values from conditional logistic regression (Supplementary Data 5) with line traversing the median. Boxes: center lines, median; box limits, upper and lower quartiles; whiskers, values within 1.5 × IQR of the top and bottom quartiles. X-axis IA = Islet Autoimmunity time of seroconversion.

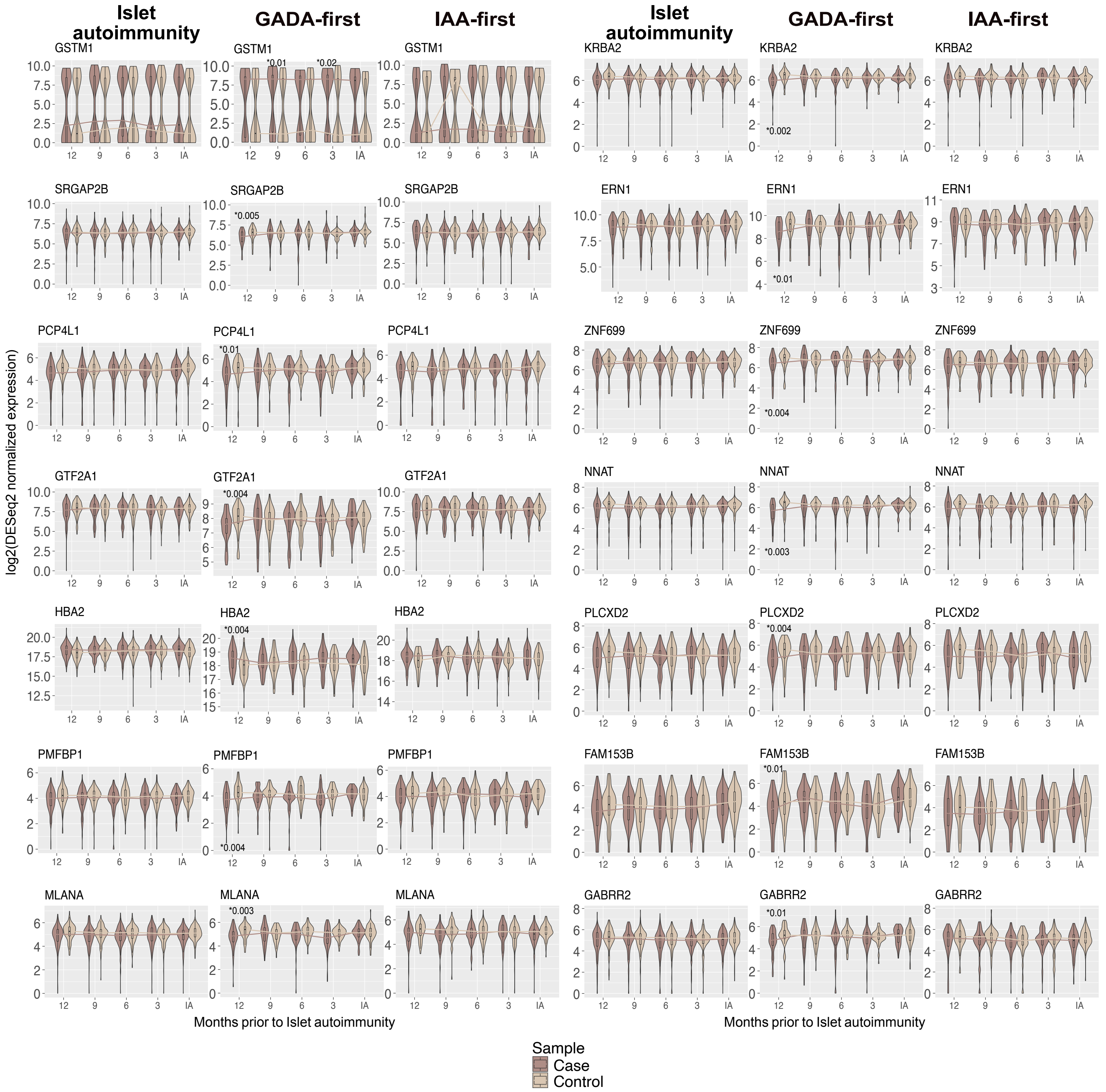

Supplementary Fig. 6: Temporal expression of the genes selected with additional criteria in GADA-first
Violin plots of the selected differentially expressed genes with inclusion of temporal slope changes and adjustment for multiple timepoints from IAb conversion to 12 months prior relative to all 3 islet autoimmunity patterns. Figure shows the 14 genes tested from GADA-first pattern in all three cohorts (full islet autoimmunity (n=168), GADA 1st (n=55) and IAA first (n=85)) and the corresponding significant p-values from conditional logistic regression (Supplementary Data 5) with line traversing the median. Boxes: center lines, median; box limits, upper and lower quartiles; whiskers, values within 1.5 × IQR of the top and bottom quartiles. X-axis IA = Islet Autoimmunity time of seroconversion.

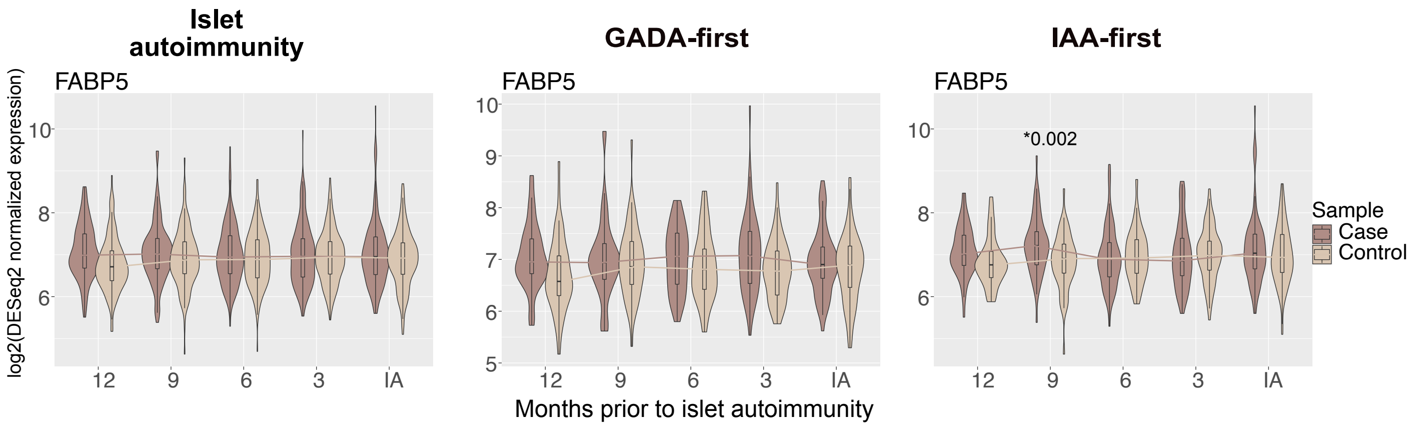

Supplementary Fig. 7: Temporal expression of the genes selected with additional criteria in IAA-first
Violin plots of the selected differentially expressed genes with inclusion of temporal slope changes and adjustment for multiple timepoints from IAb conversion to 12 months prior relative to all 3 islet autoimmunity patterns. Figure has 1 gene selected from IAA-first shown in all three cohorts (full islet autoimmunity (n=168), GADA 1st (n=55) and IAA first (n=85)) and the corresponding significant p-value from conditional logistic regression (Supplementary Data 5) with line traversing the median. Boxes: center lines, median; box limits, upper and lower quartiles; whiskers, values within 1.5 × IQR of the top and bottom quartiles. X-axis IA = Islet Autoimmunity time of seroconversion.

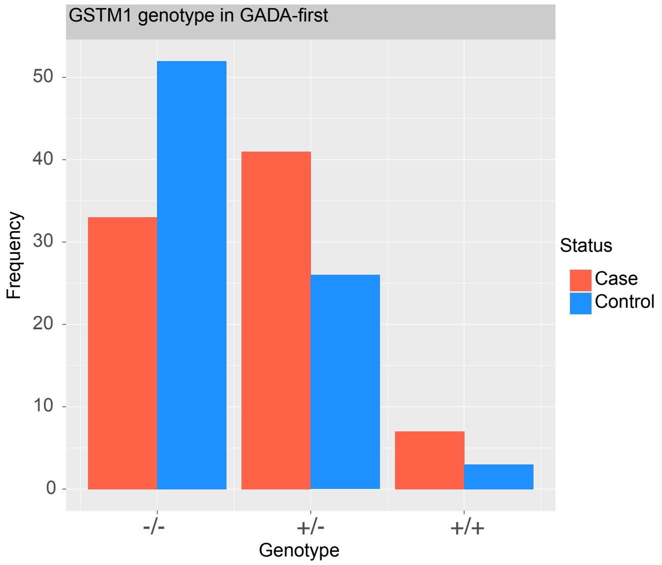

Supplementary Fig. 8: Frequencies of GSTM1 genotype in GADA-first case and control children
Barplot shows how the frequencies of genotypes in the GADA-first cohort differ for cases (red, n=81) and matched controls (blue, n=81). More controls harbor the homozygous deletion and diploid genotype was found more often in cases. Genotype is indicated as: +/+ is for diploid, +/- for hemizygous deletion, and -/- for homozygous deletion.

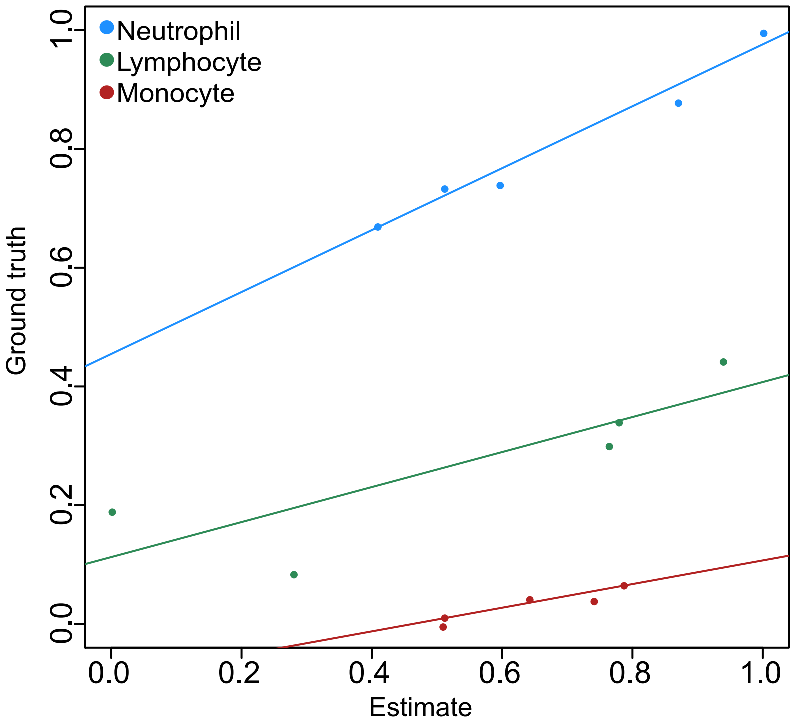

Supplementary Fig. 9: Validation of the cell-type deconvolution method
Assessment of the deconvolution method was performed using a validation reference consisting of five whole blood RNAseq samples with known cell type fractions from Gene Expression Omnibus (accession GSE60424). The deconvoluted cell type coefficients yielded 0.920 Pearson correlation with the ground truth cell type proportions.

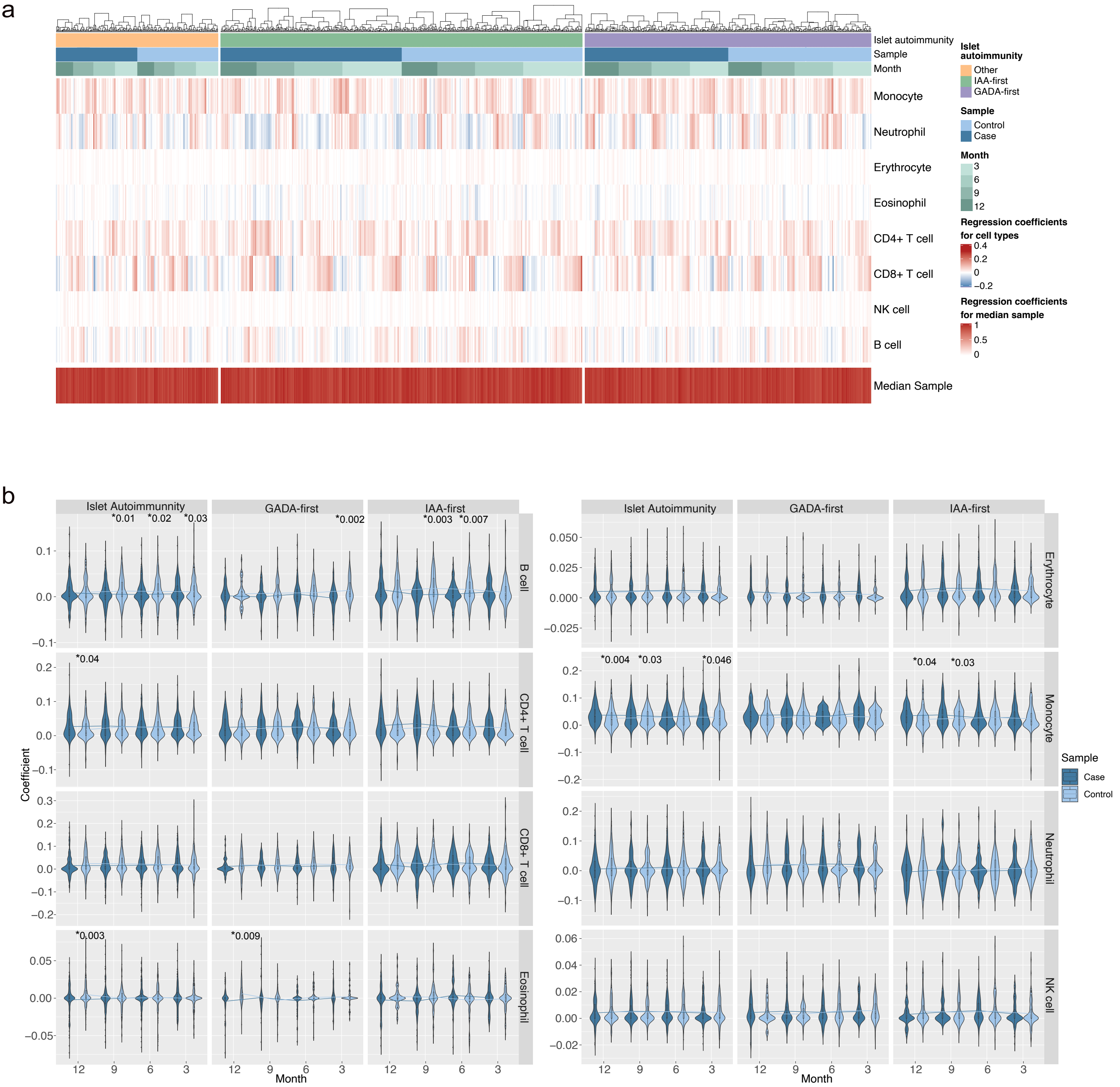

Supplementary Fig. 10: Cell type regression coefficients show differing temporal patterns between the different Islet Autoimmunity groups

(a) Expression patterns are distinct within immune cell type across IAA-first and GADA-first islet autoimmunity groups. Supporting Fig. 2d, Monocytes have higher coefficient values in cases particularly in IAA-first (middle shown in light green). Interestingly, CD4 and CD8 mostly have alternating odds ratios from 9+ months prior to seroconversion as listed in Supplementary Data 6. (b) Temporal patterns of the cell type coefficients, results significant in conditional logistic regression with p-value < 0.05 are marked with an asterisk (*) followed by the corresponding p-value. The line indicates the median, with boxes center lines, median; box limits, upper and lower quartiles; whiskers, values within 1.5 × IQR of the top and bottom quartiles.

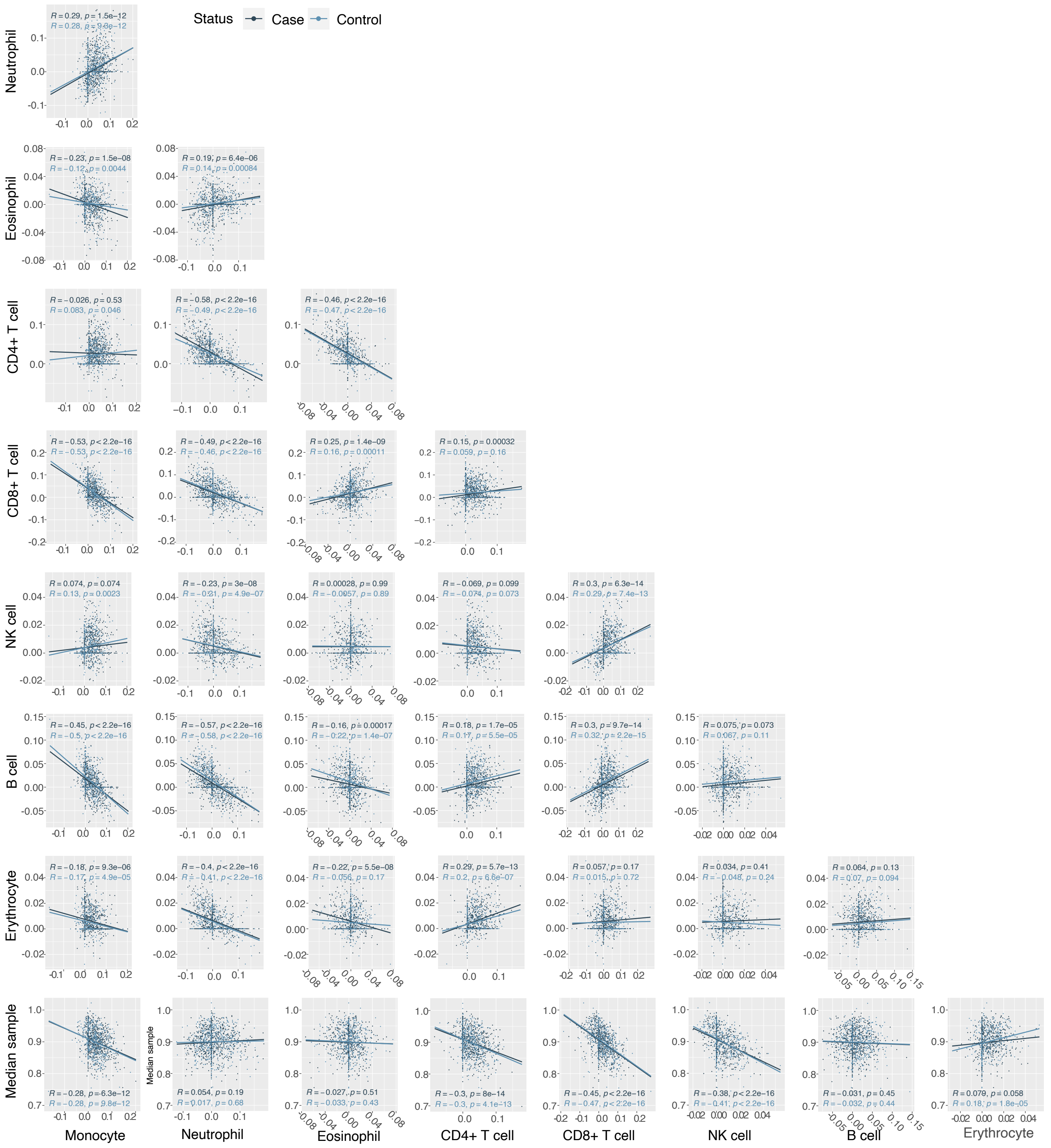

Supplementary Fig. 11: Pairwise associations between the cell type coefficients estimated from the transcriptome

Scatterplots to illustrate pairwise associations between the inferred cell type coefficients. Data is shown to support the interpretation of cell type coefficients as compositional nature of the deconvolution may introduce dependencies between cell types. Figure shows Pearson correlation coefficients (R) and their corresponding p-values (p).

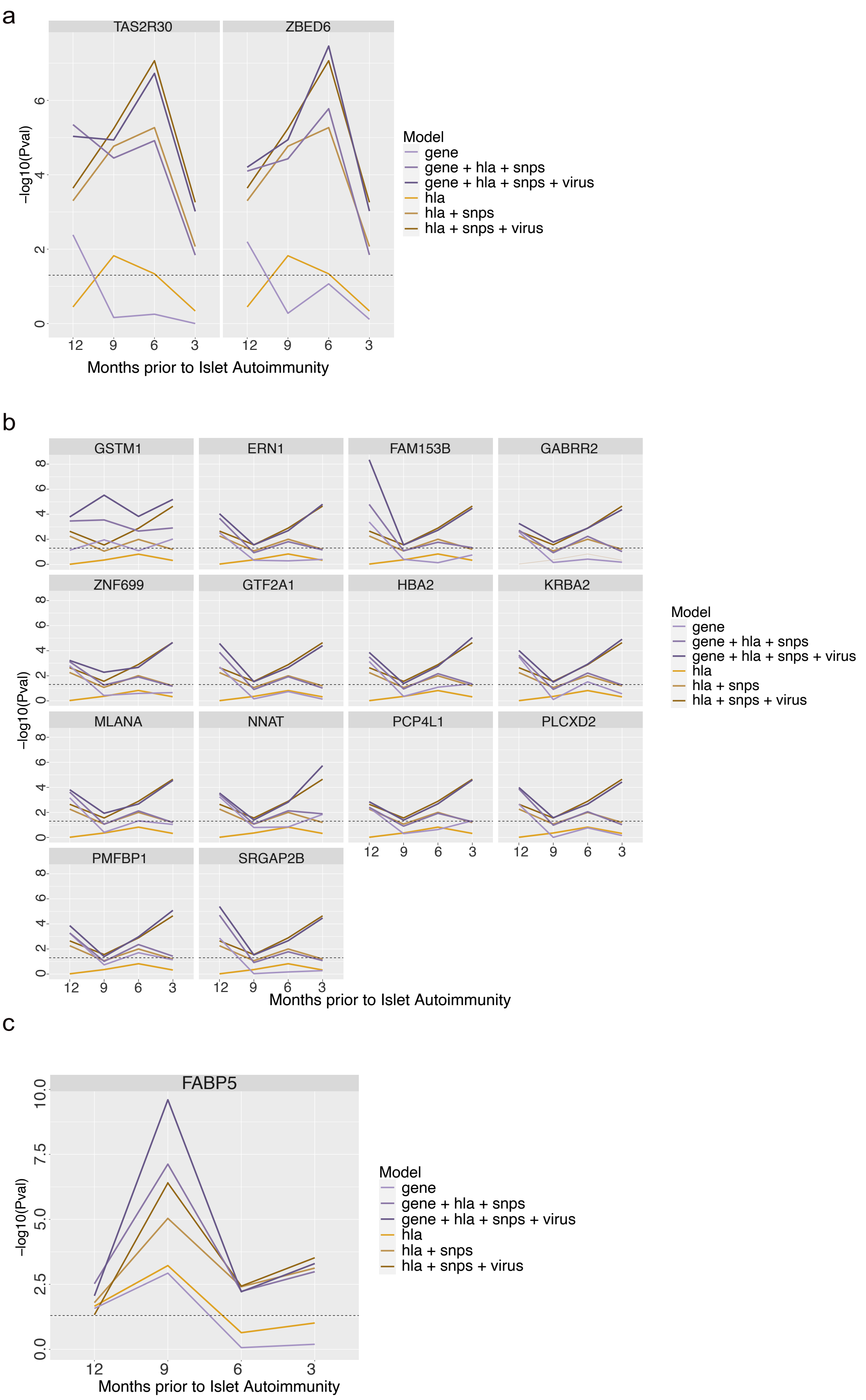

Supplementary Fig. 12: Results from Likelihood Ratio Test support the inclusion of transcriptomic data

Lineplot shows that HLA, SNP and Virus Likelihood ratio test p-values are consistently better with the inclusion of transcriptomic data for the selected genes in the (a) full islet autoimmunity (b) GADA-first and (c) IAA-first cohorts (Supplementary Data 8a-c).

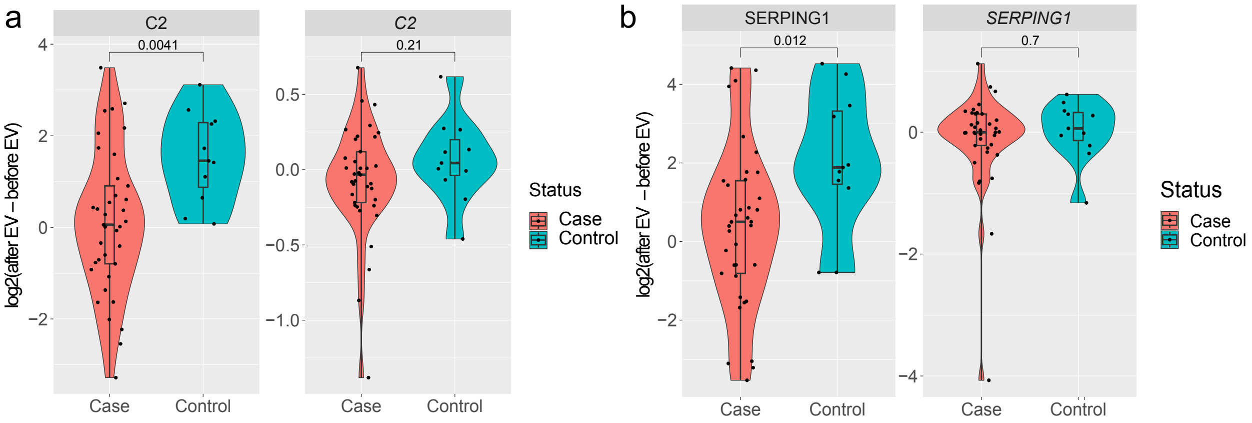

Supplementary Fig. 13: Gene expression and protein abundance show similar trend in case and control children at EV infection

Violinplots of log fold change (LFC) of the EV genes with matched protein data show a similar trend in case and control children. Gene is shown on the left, and protein on the right. Boxplot center lines, median; box limits, upper and lower quartiles; whiskers, values within 1.5 × IQR of the top and bottom quartiles. Statistic is derived with two-sided Wilcoxon rank-sum test. (a) C2 LFC for gene in case (n=37) and control (n=11) is 0.056 and 1.45 and for protein -0.034 and 0.044, in the same subjects respectively. (b) SERPING1 LFC for gene in case (n=37) and control (n=11) samples is 0.50 and 1.88, and for protein -0.002 and 0.062, respectively.

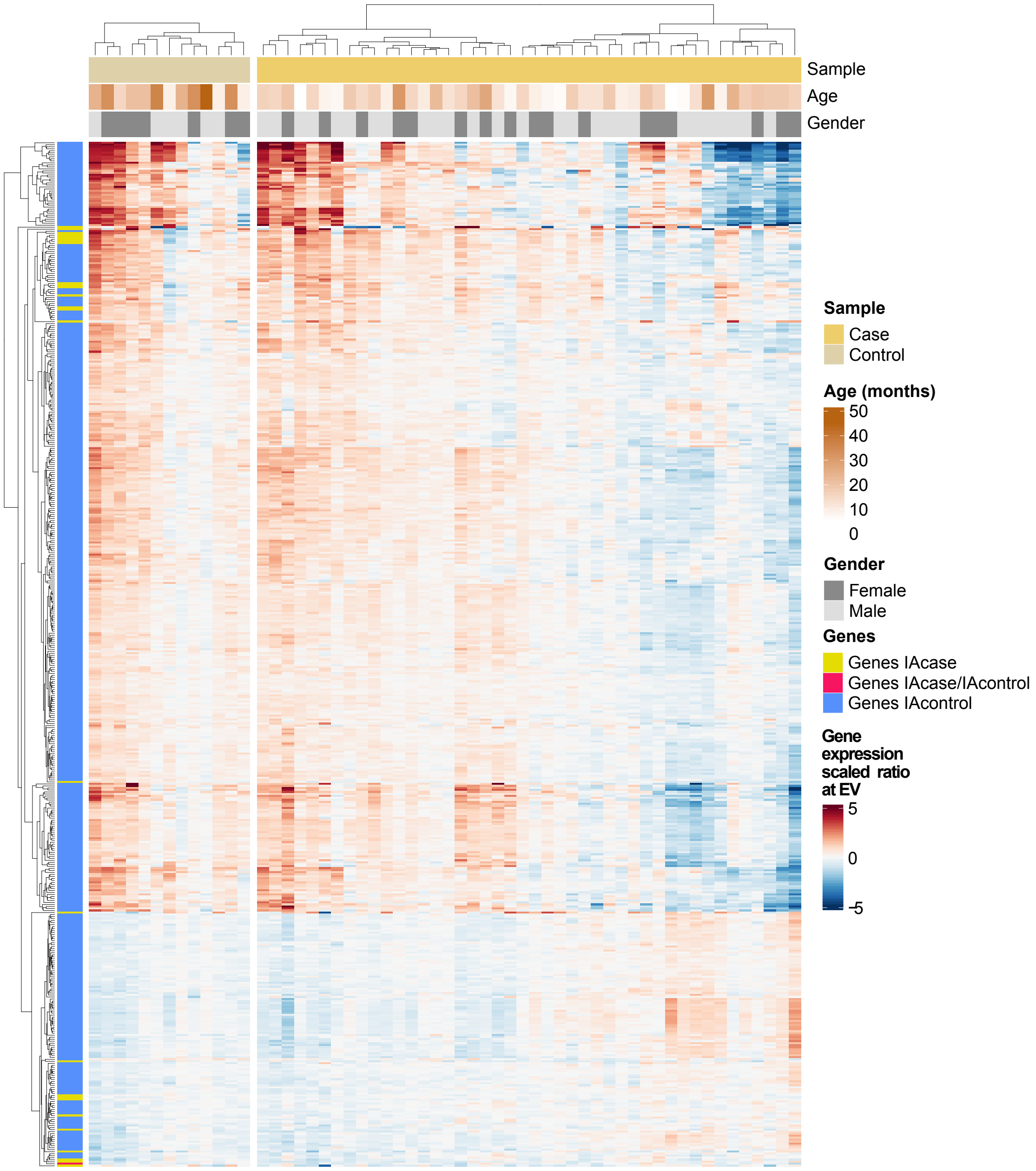

Supplementary Fig. 14: Heatmap of all the genes detected at EV in both case and control children

Distinct clusters are detected between after and prior to the initial EV infections of 483 selected genes (Supplementary Data 10) in case and control subjects. Samples with lowest gene profiles (ratio of after and prior to EV) are shown in blue and are clustered in the seroconverted children after infection.

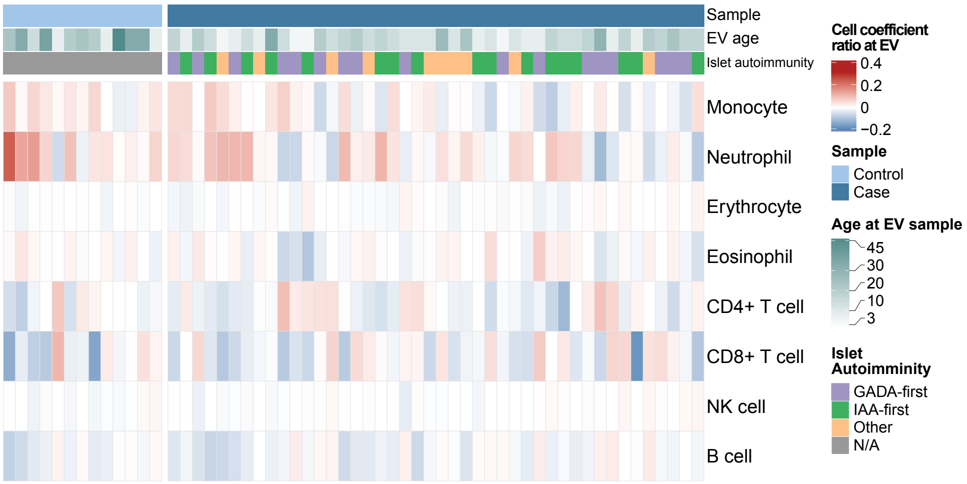

Supplementary Fig. 15: Heatmap shows immune cell type patterns before and after EV

Cell type coefficient patterns in case and control samples before and after the initial EV infection. The sample ratio patterns show negative correlation associations between monocyte and B cell as well as alternating values between CD4+ and CD8+ T cell types in seroconverted subjects. Sample order is the same as in Fig. 3a enabling comparison to gene expression patterns.

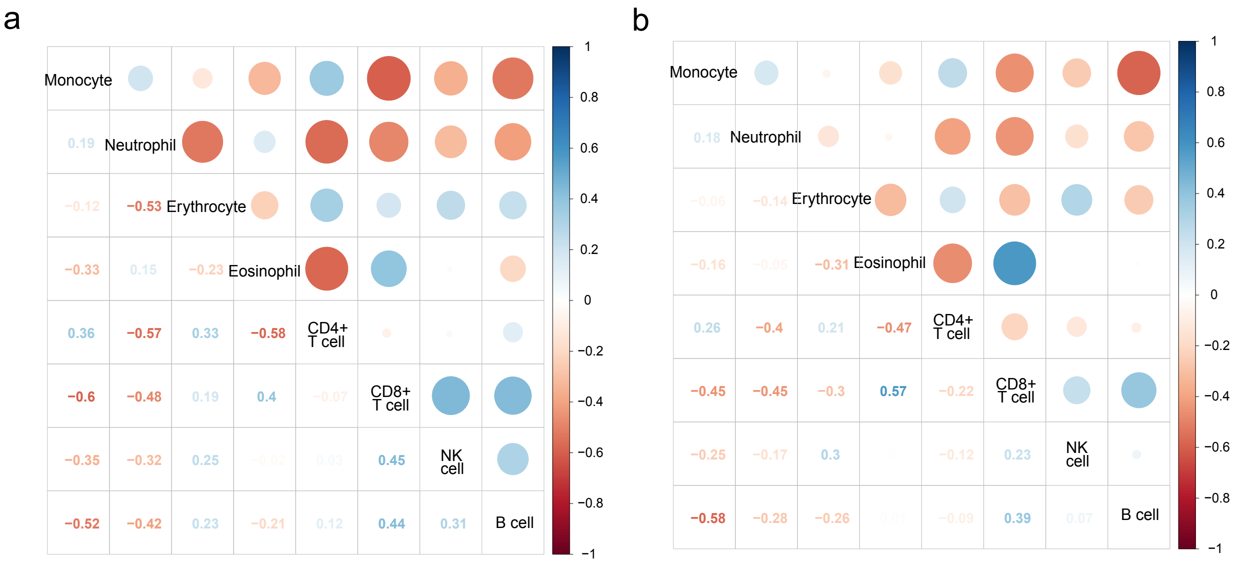

Supplementary Fig. 16: Correlation map of the cell types before and after HAdV
Correlation map between different immune cell types in control (n=74) (a) and case (n=88) (b) children based on the samples taken before and after the initial Human Adenovirus (HAdV) infection. Contrary to EV (Fig. 3d-e), the negative correlation between Neutrophils and CD8+ T cells was similar in both cases and controls (-0.48 in controls (a), -0.45 in cases (b)). Correlation coefficients were derived using the Pearson method

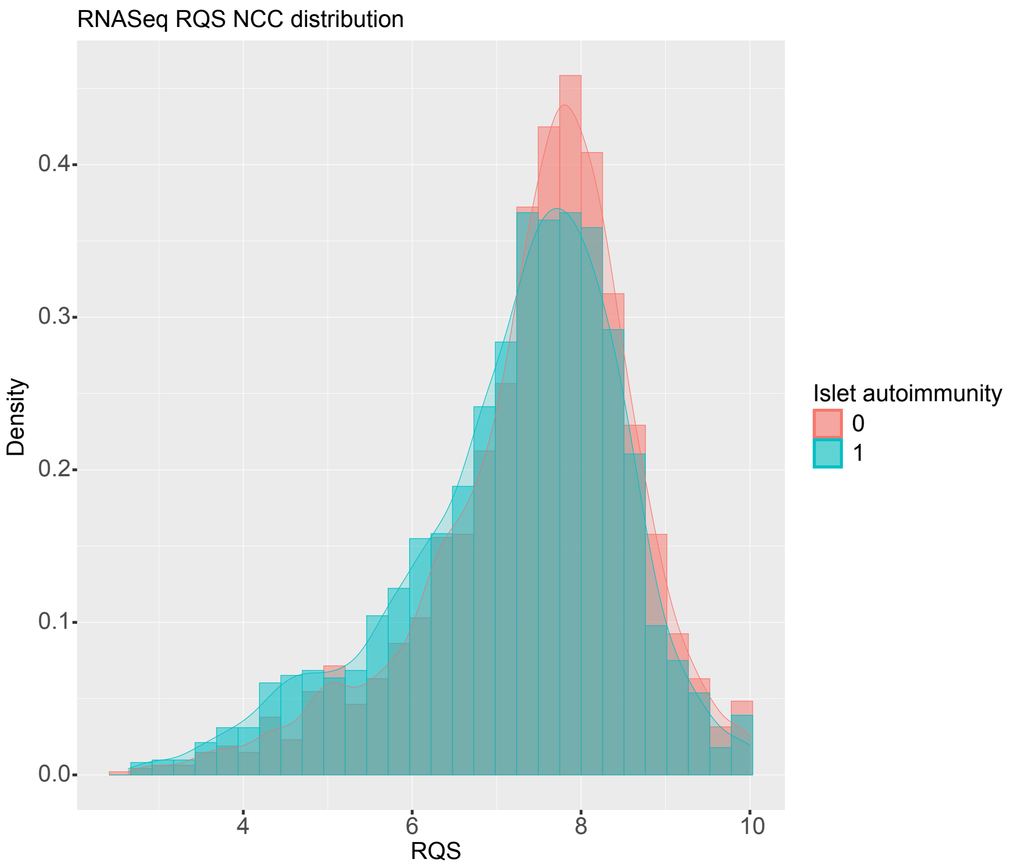

Supplementary Fig. 17: Histogram of RNAseq Quality Score distribution in the data

The histograms of the full islet autoimmunity NCC1 cohort RNAseq Quality Scores (RQS) show similar density distributions between islet autoimmunity cases and controls. Of all the samples >90% have RQS > 5.5 (median 7.54, IQR 6.67-8.16).

The TEDDY Study Group

Colorado Clinical Center: Marian Rewers, M.D., Ph.D., PI[1,4,6,9,10], Kimberly Bautista[11], Judith Baxter[8,911], Daniel Felipe-Morales, Brigitte I. Frohnert, M.D., Ph.D.[2,13], Marisa Stahl, M.D.[12], Isabel Flores Garcia, Patricia Gesualdo[2,6,11,13], Sierra Hays, Michelle Hoffman[11,12,13], Rachel Karban[11], Edwin Liu, M.D.[12], Leila Loaiza Jill Norris, Ph.D.[2,3,11], Holly O'Donnell, Ph.D.[8], Loana Thorndahl, Andrea Steck, M.D.[3,13], Kathleen Waugh[6,7,11]. University of Colorado, Anschutz Medical Campus, Barbara Davis Center for Childhood Diabetes, Aurora, CO, USA.

Finland Clinical Center: Jorma Toppari, M.D., Ph.D., PI[¥^1,4,10,13], Olli G. Simell, M.D., Ph.D., Annika Adamsson, Ph.D.[^11], Suvi Ahonen[*±§], Mari Åkerlund[*±§], Sirpa Anttila[μ¤], Leena Hakola[*±], Anne Hekkala, M.D.[μ¤], Tiia Honkanen[μ¤], Heikki Hyöty, M.D., Ph.D.[*±6], Jorma Ilonen, M.D., Ph.D.[¥3], Sanna Jokipuu[^], Taru Karjalainen[μ¤], Leena Karlsson[^], Jukka Kero M.D., Ph.D.[¥^3, 13], Jaakko J. Koskenniemi M.D., Ph.D.[¥^], Miia Kähönen[μ¤11,13], Mikael Knip, M.D., Ph.D.[*±], Minna-Liisa Koivikko[μ¤], Katja Kokkonen[*±], Merja Koskinen[*±], Mirva Koreasalo[*±§2], Kalle Kurppa, M.D., Ph.D.[*±12], Salla Kuusela, M.D. [μ¤], Jarita Kytölä[*±], Jutta Laiho, Ph.D.[*6], Tiina Latva-aho[μ¤], Siiri Leisku[*±], Laura Leppänen[^], Katri Lindfors, Ph.D.[*12], Maria Lönnrot, M.D., Ph.D.[*±6], Elina Mäntymäki[^], Markus Mattila[*±], Maija Miettinen[§2], Teija Mykkänen[μ¤], Tiina Niininen[±*11], Sari Niinistö[§2], Noora Nurminen[*±], Sami Oikarinen, Ph.D.[*±6], Hanna-Leena Oinas[*±], Paula Ollikainen[μ¤], Zhian Othmani[¥], Sirpa Pohjola [μ¤], Solja Raja-Hanhela[μ¤], Jenna Rautanen[±§], Anne Riikonen[*±§2], Minna Romo[^], Juulia Rönkä[μ¤], Nelli Rönkä[μ¤], Satu Simell, M.D., Ph.D.[¥12], Päivi Tossavainen, M.D.[μ¤], Mari Vähä-Mäkilä[¥], Eeva Varjonen[^11], Riitta Veijola, M.D., Ph.D.[μ¤13], Irene Viinikangas[μ¤], Silja Vilmi[μ¤], Suvi M. Virtanen, M.D., Ph.D.[*±§2]. [¥]University of Turku, Turku, Finland, *Tampere University, Tampere, Finland, [μ]University of Oulu, Oulu, Finland, [^]Turku University Hospital, Hospital District of Southwest Finland, Turku, Finland, [±]Tampere University Hospital, Tampere, Finland, [¤]Oulu University Hospital, Oulu, Finland, §Finnish Institute for Health and Welfare, Helsinki, Finland.

Georgia/Florida Clinical Center: Richard McIndoe, Ph.D., PI[^4,10], Desmond Schatz, M.D.[*4,7,8], Diane Hopkins[^11], Michael Haller, M.D.[*13], Risa Bernard[^11], Melissa Gardiner[^11], Ashok Sharma, Ph.D.[^], Laura Jacobsen, M.D.[*13], Jennifer Hosford[^], Kennedy Petty[^], Leah Myers[^], Chelsea Salmon*. [^]Center for Biotechnology and Genomic Medicine, Augusta University, Augusta, GA, USA. *University of Florida, Pediatric Endocrinology, Gainesville, FL, USA.

Germany Clinical Center: Anette G. Ziegler, M.D., PI[1,3,4,10], Ezio Bonifacio Ph.D.*, Cigdem Gezginci, Willi Grätz, Anja Heublein, Eva Hohoff[¥2], Sandra Hummel, Ph.D.[2], Annette Knopff[7], Melanie Köger, Sibylle Koletzko, M.D.[¶12], Claudia Ramminger[11], Roswith Roth, Ph.D.[8], Jennifer Schmidt, Marlon Scholz, Joanna Stock[8,11,13], Katharina Warncke, M.D.[13], Lorena Wendel, Christiane Winkler, Ph.D.[2,11]. Forschergruppe Diabetes e.V. and Institute of Diabetes Research, Helmholtz Zentrum München, Forschergruppe Diabetes, and Klinikum rechts der Isar, Technische Universität München, Neuherberg, Germany. *Center for Regenerative Therapies, TU Dresden, Dresden, Germany, [¶]Dr. von Hauner Children's Hospital, Department of Gastroenterology, Ludwig Maximillians University Munich, Munich, Germany, [¥]University of Bonn, Department of Nutritional Epidemiology, Bonn, Germany.

Sweden Clinical Center: Åke Lernmark, Ph.D., PI[1,3,4,5,6,8,9,10], Daniel Agardh, M.D., Ph.D.[6,12], Carin Andrén Aronsson, Ph.D.[2,11,12], Rasmus Bennet, Corrado Cilio, Ph.D., M.D.[6], Susanne Dahlberg, Ulla Fält, Malin Goldman Tsubarah, Emelie Ericson-Hallström, Lina Fransson, Emina Halilovic, Gunilla Holmén, Susanne Hyberg, Berglind Jonsdottir, M.D., Ph.D.[11], Naghmeh Karimi, Helena Elding Larsson, M.D., Ph.D.[6,13], Marielle Lindström, Markus Lundgren, M.D., Ph.D.[13], Marlena Maziarz, Ph.D., Jessica Melin[11], Caroline Nilsson, Kobra Rahmati, Anita Ramelius, Falastin Salami, Ph.D., Anette Sjöberg, Evelyn Tekum Amboh Carina Törn, Ph.D.[3], Ulrika Ulvenhag, Terese Wiktorsson, Åsa Wimar[13]. Lund University, Lund, Sweden.

Washington Clinical Center: William A. Hagopian, M.D., Ph.D., PI[1,3,4,6,7,10,12,13], Michael Killian[6,7,11,12], Claire Cowen Crouch[11,13], Jennifer Skidmore[2], Trevor Bender, Megan Llewellyn, Cody McCall, Arlene Meyer, Jocelyn Meyer, Denise Mulenga[11], Nole Powell, Jared Radtke, Shreya Roy, Preston Tucker. Pacific Northwest Research Institute, Seattle, WA, USA.

Pennsylvania Satellite Center: Dorothy Becker, M.D., Margaret Franciscus, MaryEllen Dalmagro-Elias Smith[2], Ashi Daftary, M.D., Mary Beth Klein, Chrystal Yates. Children's Hospital of Pittsburgh of UPMC, Pittsburgh, PA, USA.

<u>Data Coordinating Center:</u> Jeffrey P. Krischer, Ph.D., PI[1,4,5,9,10], Rajesh Adusumali, Sarah Austin-Gonzalez, Maryouri Avendano, Sandra Baethke, Brant Burkhardt, Ph.D.[6], Martha Butterworth[2], Nicholas Cadigan, Joanna Clasen, Kevin Counts, Laura Gandolfo, Jennifer Garmeson, Veena Gowda, Christina Karges, Shu Liu, Xiang Liu, Ph.D.[2,3,8,13], Kristian Lynch, Ph.D. [6,8], Jamie Malloy, Lazarus Mramba, Ph.D.[2], Cristina McCarthy[11], Jose Moreno, Hemang M. Parikh, Ph.D.[3,8], Cassandra Remedios, Chris Shaffer, Susan Smith[11], Noah Sulman, Ph.D., Roy Tamura, Ph.D.[1,2,11,12,13], Dena Tewey, Henri Thuma, Michael Toth, Ulla Uusitalo, Ph.D.[2], Kendra Vehik, Ph.D.[4,5,6,8,13], Ponni Vijayakandipan, Melissa Wroble, Jimin Yang, Ph.D., R.D.[2], Kenneth Young, Ph.D. *Past staff: Michael Abbondondolo, Lori Ballard, Rasheedah Brown, David Cuthbertson, Stephen Dankyi, Christopher Eberhard, Steven Fiske, David Hadley, Ph.D., Kathleen Heyman, Belinda Hsiao, Francisco Perez Laras, Hye-Seung Lee, Ph.D., Qian Li, Ph.D., Colleen Maguire, Wendy McLeod, Aubrie Merrell, Steven Meulemans, Ryan Quigley, Laura Smith, Ph.D.* University of South Florida, Tampa, FL, USA.

<u>Autoantibody Reference Laboratories:</u> Liping Yu, M.D.[^5], Dongmei Miao, M.D.[^], Kathleen Gillespie*[5], Kyla Chandler*, Ilana Kelland*, Yassin Ben Khoud*, Matthew Randell *. [^]Barbara Davis Center for Childhood Diabetes, University of Colorado Denver, *Bristol Medical School, University of Bristol, UK.

<u>Genetics Laboratory:</u> Stephen S. Rich, Ph.D.[3], Wei-Min Chen, Ph.D.[3], Suna Onengut-Gumuscu, Ph.D.[3], Emily Farber, Rebecca Roche Pickin, Ph.D., Jonathan Davis, Jordan Davis, Dan Gallo, Jessica Bonnie, Paul Campolieto. Center for Public Health Genomics, University of Virginia, Charlottesville, VA, USA.

<u>HLA Reference Laboratory:</u> William Hagopian[3], M.D., Ph.D., Jared Radtke, Preston Tucker. Pacific Northwest Research Institute, Seattle, WA, USA. (Previously Henry Erlich, Ph.D.[3], Steven J. Mack, Ph.D., Anna Lisa Fear. Center for Genetics, Children's Hospital Oakland Research Institute.)

<u>Metagenomics and Microbiome Laboratory:</u> Joseph F. Petrosino, Ph.D.[6*], Nadim J. Ajami, Ph.D.[*], Richard E. Lloyd, Ph.D.[6*], Matthew C. Ross, Ph.D.[*], Jacqueline L. O'Brien, Ph.D.[*], Diane S. Hutchinson, Ph.D.[*], Daniel P. Smith, Ph.D.[*], Matthew C. Wong[*], Xianjun Tian, Ph.D.[*], Tulin Ayvaz[*], Auriole Tamegnon[*], Nguyen Truong[*], Hannah Moreno[*], Lauren Riley[*], Eduardo Moreno[*], Tonya Bauch[*], Lenka Kusic[*], Ginger Metcalf[^], Donna Muzny[^], HarshaVArdhan Doddapaneni, Ph.D.[^], Richard Gibbs, Ph.D.[^]. *Alkek Center for Metagenomics and Microbiome Research, Department of Molecular Virology and Microbiology, Baylor College of Medicine, Houston, TX, USA. ^Human Genome Sequencing Center, Baylor College of Medicine, Houston, TX, USA..

<u>Repository:</u> Chris Deigan. NIDDK Biosample Repository at Fisher BioServices, Rockville, MD, USA. (Previously Ricky Schrock, Polina Malone, Sandra Ke, Niveen Mulholland, Ph.D.)

<u>Project scientist:</u> Beena Akolkar, Ph.D.[1,3,4,5,6,7,9,10]. National Institutes of Diabetes and Digestive and Kidney Diseases, Bethesda, MD, USA.

<u>Other contributors:</u> Thomas Briese, Ph.D.[6], Columbia University. Todd Brusko, Ph.D.[5], University of Florida, Gainesville, FL, USA. Teresa Buckner, Ph.D.[2], University of Northern Colorado, Greeley, CO. Suzanne Bennett Johnson, Ph.D.[8,11], Florida State University, Tallahassee, FL, USA. Eoin McKinney, Ph.D.[5], University of Cambridge, Cambridge, UK. Tomi Pastinen, M.D., Ph.D.[5], The Children's Mercy Hospital, Kansas City, MO, USA. Steffen Ullitz Thorsen, M.D., Ph.D.[2], Department of Clinical Immunology, University of Copenhagen, Copenhagen, Denmark, and Department of Pediatrics and Adolescents, Copenhagen University Hospital, Herlev, Denmark. Eric Triplett, Ph.D.[6], University of Florida, Gainesville, FL, USA.

Committees:
[1]Ancillary Studies, [2]Diet, [3]Genetics, [4]Human Subjects/Publicity/Publications, [5]Immune Markers, [6]Infectious Agents, [7]Laboratory Implementation, [8]Psychosocial, [9]Quality Assurance, [10]Steering, [11]Study Coordinators, [12]Celiac Disease, [13]Clinical Implementation.
