# Supplementary material for: Distinct transcriptomic profiles in children prior to the appearance of type 1 diabetes-linked islet autoantibodies and following enterovirus infection
Source: Nat Commun. 2023 Nov 22;14:7630. doi: 10.1038/s41467-023-42763-9 (PMC10665402; doi:10.1038/s41467-023-42763-9)
Supplement: Supplementary file 3 — Description of Additional Supplementary Files [file 41467_2023_42763_MOESM3_ESM.pdf]

Description of Additional Supplementary Files

Supplementary Data 1
Description: Demographics of TEDDY NCC1 Islet Autoimmunity

Supplementary Data 2
Description: Differentially expressed genes with their log fold changes and adjusted p-values

Supplementary Data 3
Description: Enriched pathways for the differentially expressed genes

Supplementary Data 4
Description: Genes selected with additional temporal criteria

Supplementary Data 5
Description: Conditional logistic regression results for the selected genes

Supplementary Data 6
Description: Conditional logistic regression results for the cell types

Supplementary Data 7
Description: Conditional logistics regression results for viral infections

Supplementary Data 8
Description: Likelihood Ratio Test Results

Supplementary Data 9
Description: Viral exposures in subjects in EV analysis

Supplementary Data 10
Description: Differentially expressed genes at EV infection

Supplementary Data 11
Description: Enriched pathways at EV infection

Supplementary Data 12
Description: Differentially expressed genes at hAdV infection

Supplementary Data 13
Description: Cell type correlation between samples taken before and after EV

Supplementary Data 14
Description: Conditional logistics results for cell types at EV

Supplementary Data 15
Description: Accession IDs for external data used in the deconvolution
