# Supplementary material for: Distinct transcriptomic profiles in children prior to the appearance of type 1 diabetes-linked islet autoantibodies and following enterovirus infection
Source: Nat Commun. 2023 Nov 22;14:7630. doi: 10.1038/s41467-023-42763-9 (PMC10665402; doi:10.1038/s41467-023-42763-9)
Supplement: Supplementary file 5 — Reporting Summary [file 41467_2023_42763_MOESM5_ESM.pdf]

nature portfolio

Corresponding author(s): Matti Nykter
Kirsi Rautajoki
Heikki Hyöty

Last updated by author(s): Sep 22, 2023

Reporting Summary

Nature Portfolio wishes to improve the reproducibility of the work that we publish. This form provides structure for consistency and transparency in reporting. For further information on Nature Portfolio policies, see our Editorial Policies and the Editorial Policy Checklist.

Statistics

For all statistical analyses, confirm that the following items are present in the figure legend, table legend, main text, or Methods section.

n/a	Confirmed	
☐	☒	The exact sample size (n) for each experimental group/condition, given as a discrete number and unit of measurement
☐	☒	A statement on whether measurements were taken from distinct samples or whether the same sample was measured repeatedly
☐	☒	The statistical test(s) used AND whether they are one- or two-sided<br>*Only common tests should be described solely by name; describe more complex techniques in the Methods section.*
☐	☒	A description of all covariates tested
☐	☒	A description of any assumptions or corrections, such as tests of normality and adjustment for multiple comparisons
☐	☒	A full description of the statistical parameters including central tendency (e.g. means) or other basic estimates (e.g. regression coefficient) AND variation (e.g. standard deviation) or associated estimates of uncertainty (e.g. confidence intervals)
☐	☒	For null hypothesis testing, the test statistic (e.g. F, t, r) with confidence intervals, effect sizes, degrees of freedom and P value noted<br>*Give P values as exact values whenever suitable.*
☒	☐	For Bayesian analysis, information on the choice of priors and Markov chain Monte Carlo settings
☒	☐	For hierarchical and complex designs, identification of the appropriate level for tests and full reporting of outcomes
☐	☒	Estimates of effect sizes (e.g. Cohen's d, Pearson's r), indicating how they were calculated

Our web collection on statistics for biologists contains articles on many of the points above.

Software and code

Policy information about availability of computer code

Data collection	No software was used for data collection
Data analysis	Analysis was performed using open source tools and libraries. Custom scripts for key analysis steps are available on the GitHub repository https://github.com/NykterLab/TEDDY_IA (10.5281/zenodo.8345041). Vipie virome web application scripts are available on https://sourceforge.net/projects/vipie/ and hosted on http://vipie.rd.tuni.fi/vipie/index.html. Custom offline Vipie (Vipie: https://sourceforge.net/p/vipie/code/HEAD/tree/scripts/) was used to generate the virome data. The enterovirus capsid libraries are available upon request from the Tampere Virology Group (https://research.tuni.fi/virology/).<br><br>Statistical testing of NCC was performed with Conditional logistic regression (CLR) as part of the survival package from CRAN. Other statistical testing and plotting are performed using R 4.0 and relevant packages.<br><br>RNA-Seq differential expression analysis were performed using DeSEQ2.<br>Cell type deconvolution was performed using custom algorithm based on elasticnet, available at above mentioned github repository.<br>Correlation was visualized and tested using corrplot and cor.mtest

For manuscripts utilizing custom algorithms or software that are central to the research but not yet described in published literature, software must be made available to editors and reviewers. We strongly encourage code deposition in a community repository (e.g. GitHub). See the Nature Portfolio guidelines for submitting code & software for further information.

Data

Policy information about availability of data

All manuscripts must include a data availability statement. This statement should provide the following information, where applicable:

- Accession codes, unique identifiers, or web links for publicly available datasets
- A description of any restrictions on data availability
- For clinical datasets or third party data, please ensure that the statement adheres to our policy

The TEDDY sequencing data used in this study have been deposited in the dbGaP database under accession code phs001442.v4.p3 [https://www.ncbi.nlm.nih.gov/projects/gap/cgi-bin/study.cgi?study_id=phs001442.v4.p3]. The TEDDY omics data are available under restricted access for sensitivity reasons, access can be obtained by request through dbGaP. The results data including log fold changes and cell type coefficients generated in this study are provided in the Supplementary Information/Source Data file. The mass spectrometry raw data used in this study are available in the MassIVE database under accession codes MSV000091560 (untargeted proteomics) and MSV000091562 (targeted proteomics) [https://massive.ucsd.edu/]. Clinical metadata analyzed for the current study is available in the NIDDK Central Repository [https://repository.niddk.nih.gov/studies/teddy/]. The deconvolution validation data used in this study is available in the Gene Expression Omnibus (GEO) under accession code GSE60424 [https://www.ncbi.nlm.nih.gov/geo/query/acc.cgi?acc=GSE60424]. The reference cell type data used in deconvolution analysis are available in the GEO database under accession codes GSM971331 [https://www.ncbi.nlm.nih.gov/geo/query/acc.cgi?acc=GSM971331] , GSM823383 [https://www.ncbi.nlm.nih.gov/geo/query/acc.cgi?acc=GSM823383], GSM1060237 [https://www.ncbi.nlm.nih.gov/geo/query/acc.cgi?acc=GSM1060237], GSM3319903 [https://www.ncbi.nlm.nih.gov/geo/query/acc.cgi?acc=GSM3319903], GSM1576438 [https://www.ncbi.nlm.nih.gov/geo/query/acc.cgi?acc=GSM1576438], GSM986103 [https://www.ncbi.nlm.nih.gov/geo/query/acc.cgi?acc=GSM986103], GSM996197 [https://www.ncbi.nlm.nih.gov/geo/query/acc.cgi?acc=GSM996197], GSM996200 [https://www.ncbi.nlm.nih.gov/geo/query/acc.cgi?acc=GSM996200], GSM3039712 [https://www.ncbi.nlm.nih.gov/geo/query/acc.cgi?acc=GSM3039712], GSM3039716 [https://www.ncbi.nlm.nih.gov/geo/query/acc.cgi?acc=GSM3039716], GSM3039720 [https://www.ncbi.nlm.nih.gov/geo/query/acc.cgi?acc=GSM3039720] and GSM1657640 [https://www.ncbi.nlm.nih.gov/geo/query/acc.cgi?acc=GSM1657640].

Human research participants

Policy information about studies involving human research participants and Sex and Gender in Research.

Reporting on sex and gender	Study cohort has been matched for biological sex, clinical site and family history with T1D. Thus, sex related aspects are not studied.
Population characteristics	Children were a mean(stddev) of 2.1(1.2) years of age in the IA case-control and 2.7(1.4) years of age in the T1D case-control. Children samples were obtained from six geographical locations (Finland, Germany, Sweden in Europe and Washington, Colorado and Georgia in the United States). These children are at high HLA genetic risk for developing IA or T1D, with half of the cases for IA or T1D and the other half controls (Supplementary Table 1).
Recruitment	Children were recruited based on specific type 1 diabetes risk human leukocyte antigen (HLA) genotypes and family history of T1D risk. Recruitment began in September of 2004 and was completed in February 2010. Six clinical centers in the USA (Colorado, Washington, and Florida/Georgia) and Europe (Germany, Sweden, and Finland) randomly HLA-screened 424,788 children at birth in hospitals in the four countries. A total of 418,367 general population infants were screen, of which 20,152 (4.8%) were eligible, and 1,437 of the 6,421 screened infants (22.4%) with a first-degree relative with type 1 diabetes were eligible. There were 8,676 children enrolled as participants in the study
Ethics oversight	The TEDDY study was approved by local US Institutional Review Boards and European Ethics Committee Boards in Colorado's Colorado Multiple Institutional Review Board, Georgia's Medical College of Georgia Human Assurance Committee (2004–2010), Georgia Health Sciences University Human Assurance Committee (2011–2012), Georgia Regents University Institutional Review Board (2013–2015), Augusta University Institutional Review Board (2015–present), Florida's University of Florida Health Center Institutional Review Board, Washington state's Washington State Institutional Review Board (2004–2012) and Western Institutional Review Board (2013–present), Finland's Ethics Committee of the Hospital District of Southwest Finland, Germany's Bayerischen Landesärztekammer (Bavarian Medical Association) Ethics Committee, Sweden's Regional Ethics Board in Lund, Section 2 (2004–2012) and Lund University Committee for Continuing Ethical Review (2013–present). All parents or guardians provided written informed consent before participation in genetic screening and enrolment. The study was performed in compliance with all relevant ethical regulations. Study has been registered at clinicaltrials.gov with trial registration number NCT00279318.

Note that full information on the approval of the study protocol must also be provided in the manuscript.

Field-specific reporting

Please select the one below that is the best fit for your research. If you are not sure, read the appropriate sections before making your selection.

☒ Life sciences ☐ Behavioural & social sciences ☐ Ecological, evolutionary & environmental sciences

For a reference copy of the document with all sections, see nature.com/documents/nr-reporting-summary-flat.pdf

Life sciences study design

All studies must disclose on these points even when the disclosure is negative.

Sample size	TEDDY IA Nested Case Control (NCC) study consists of 418 pairs of children. 383 pairs had matching stool or plasma virome, and 370 had whole blood transcriptome data available. 312 IA NCC pairs had data from both data types. Transcriptome data included in total 1693 sample pairs from 370 case-control pairs over 0-12 months period prior to IA seroconversion. A total of 9911 (4536 matched) stool and 4779 (2343 matched) plasma virome samples were processed using Vipie and for Enterovirus hits, further processed with capsid (V1-V4) matching. A priori power calculations for nested-matched case–control studies show that study will have ≥80% power at a significance level of 5% to detect an OR>2.28 for the 383 pairs.
Data exclusions	For the nested case-control analysis, some samples were removed so that exactly the same number of samples was included between case and control pairs. This prevented skewing data due to the generally increased number of samples from cases and missing matched pair control data. This lack of matching stool sampling availability resulted in a 9% reduction in the number of pairs for the 1:1 viral metagenomic study for the islet autoimmunity nested-matched case-control, which left 383 pairs (n = 4,327 age matched stool samples in each group). All outcome analyses were conducted on matched-pair sample data.
Replication	Observation cohort. No replication
Randomization	Controls were matched individually to cases as described earlier. Cases were sampled based on specific case-control design (i.e., until either development of islet autoimmunity or diagnosis of T1D) and matched control samples were included up until the corresponding age of event. The experiments were not randomized, and investigators were not blinded to allocation during experiments and outcome assessment.
Blinding	TEDDY is an observational follow-up study, thus no overall blinding was used. However, the selection of samples sent processing were determined by the Data Coordinating Center (USF Health Informatics Institute, Tampa, FL) without the laboratory knowing the case-control status.

Reporting for specific materials, systems and methods

We require information from authors about some types of materials, experimental systems and methods used in many studies. Here, indicate whether each material, system or method listed is relevant to your study. If you are not sure if a list item applies to your research, read the appropriate section before selecting a response.

Materials & experimental systems

n/a	Involved in the study	
☒	☐	Antibodies
☒	☐	Eukaryotic cell lines
☒	☐	Palaeontology and archaeology
☒	☐	Animals and other organisms
☐	☒	Clinical data
☒	☐	Dual use research of concern

Methods

n/a	Involved in the study	
☒	☐	ChIP-seq
☒	☐	Flow cytometry
☒	☐	MRI-based neuroimaging

Clinical data

Policy information about clinical studies

All manuscripts should comply with the ICMJE guidelines for publication of clinical research and a completed CONSORT checklist must be included with all submissions.

Clinical trial registration	NCT00279318
Study protocol	Full protocol can be accessed at https://teddy.epi.usf.edu/documents/TEDDY_Protocol.pdf
Data collection	Six clinical research centers - three in the U.S. (Colorado, Georgia/Florida, Washington), and three in Europe (Finland, Germany, and Sweden) participated in a population-based HLA screening of newborns between 2004 and 2010. Children with high risk HLA genotypes were enrolled (n=8,676) and prospectively followed from three months of age to 15 years with study visits that include a blood draw every three months until four years and every three or six months thereafter depending on islet autoimmunity positivity. Stool samples were collected monthly from ages 3 to 48 months and then quarterly until the age of 10 years. A nested-matched case-control was conducted through risk-set sampling using metadata and islet autoimmunity sample results as of 31 May 2012. In a separate nested-matched case-control, each child diagnosed with T1D had a control selected based on their event time from birth. Metadata were collected using validated questionnaires that have been either published or extensively scrutinized by experts. TEDDY provides many tools, such as 'The TEDDY book', to the parents to assist in real-time collection of all events in their child's life to ensure bias and error are minimized.
Outcomes	Persistent confirmed autoimmunity was defined by the presence of a confirmed islet autoimmunity (glutamic acid decarboxylase (GADA), insulinoma-associated 2 (IA-2A) or insulin (IAA)) at each of the two TEDDY laboratories on two or more consecutive visits. T1D diagnosis was defined according to American Diabetes Association criteria. Discordance between cases and matched controls, both in the number of stool samples positive for each common virus were evaluated using conditional logistic regression models adjusting for HLA genotypes. We test on all IA, IAA first, as well as GADA first outcomes. We further evaluate host response upon Enterovirus (EV) exposure by identifying the first EV exposure (stool or plasma) with availability of RNASeq sample in the same month window (stool is monthly, plasma and RNASeq are quarterly) as well as a prior EV free RNASeq sample. The magnitude of the associations were assessed by odds ratios with 95% confidence intervals

nature portfolio | reporting summary
